# Contribution of Bicarbonate Assimilation to Carbon Pool Dynamics in the Deep Mediterranean Sea and Cultivation of Actively Nitrifying and CO_2_-Fixing Bathypelagic Prokaryotic Consortia

**DOI:** 10.3389/fmicb.2018.00003

**Published:** 2018-01-19

**Authors:** Violetta La Cono, Gioachino Ruggeri, Maurizio Azzaro, Francesca Crisafi, Franco Decembrini, Renata Denaro, Gina La Spada, Giovanna Maimone, Luis S. Monticelli, Francesco Smedile, Laura Giuliano, Michail M. Yakimov

**Affiliations:** ^1^Institute for Coastal Marine Environment, National Research Council, Messina, Italy; ^2^Department of Marine and Coastal Sciences, Rutgers University, New Brunswick, NJ, United States; ^3^Mediterranean Science Commission (CIESM), Monaco, Monaco; ^4^Institute of Living Systems, Immanuel Kant Baltic Federal University, Kaliningrad, Russia

**Keywords:** dark bicarbonate assimilation, anaplerotic reactions, deep-sea microbial community, mediterranean sea, ammonium-oxidizing *Thaumarchaeota*

## Abstract

Covering two-thirds of our planet, the global deep ocean plays a central role in supporting life on Earth. Among other processes, this biggest ecosystem buffers the rise of atmospheric CO_2_. Despite carbon sequestration in the deep ocean has been known for a long time, microbial activity in the meso- and bathypelagic realm via the “*assimilation of bicarbonate in the dark*” (ABD) has only recently been described in more details. Based on recent findings, this process seems primarily the result of chemosynthetic and anaplerotic reactions driven by different groups of deep-sea prokaryoplankton. We quantified bicarbonate assimilation in relation to total prokaryotic abundance, prokaryotic heterotrophic production and respiration in the meso- and bathypelagic Mediterranean Sea. The measured ABD values, ranging from 133 to 370 μg C m^−3^ d^−1^, were among the highest ones reported worldwide for similar depths, likely due to the elevated temperature of the deep Mediterranean Sea (13–14°C also at abyssal depths). Integrated over the dark water column (≥200 m depth), bicarbonate assimilation in the deep-sea ranged from 396 to 873 mg C m^−2^ d^−1^. This quantity of produced *de novo* organic carbon amounts to about 85–424% of the phytoplankton primary production and covers up to 62% of deep-sea prokaryotic total carbon demand. Hence, the ABD process in the meso- and bathypelagic Mediterranean Sea might substantially contribute to the inorganic and organic pool and significantly sustain the deep-sea microbial food web. To elucidate the ABD key-players, we established three actively nitrifying and CO_2_-fixing prokaryotic enrichments. Consortia were characterized by the co-occurrence of chemolithoautotrophic *Thaumarchaeota* and chemoheterotrophic proteobacteria. One of the enrichments, originated from Ionian bathypelagic waters (3,000 m depth) and supplemented with low concentrations of ammonia, was dominated by the *Thaumarchaeota* “low-ammonia-concentration” deep-sea ecotype, an enigmatic and ecologically important group of organisms, uncultured until this study.

## Introduction

Deep aphotic pelagic water masses represent the largest marine area comprising almost three quarters of the global oceanic volume and contain majority of the global dissolved inorganic carbon (DIC) pool (Arístegui et al., 2009; Reinthaler et al., 2010). Despite that, deep-sea environment belongs by far to the least studied ecosystems on Earth. The current database contains very few data about microbial life and steering biogeochemical processes in this oceanic interim. Dark ocean carbon budgets and, especially, prokaryotic carbon demand based on carbon flux and metabolic activity are fraught with uncertainties and discrepancies (Arístegui et al., 2009; Baltar et al., 2009; Burd et al., 2010). Until recently, the deep ocean was supposed to be exclusively heterotrophic and dependent on vertical flux and lateral advection of organic carbon either produced by phytoplankton in the sunlit surface waters or entered from the atmosphere (Duarte et al., 2013). However, recent compiled global carbon budgets and intensive local field data suggest that the estimate of metabolic activity in the dark pelagic ocean exceeds estimated inputs of organic carbon (Burd et al., 2010). In some bathypelagic waters, this imbalance between carbon supply and prokaryotic carbon demand (the sum of heterotrophic biomass production and respiration) can reach about two orders of magnitude. The apparent paradox of carbon balance in dark oceans might result from the overestimation of *in situ* metabolic activities, the underestimation of organic carbon inputs from the upper layers (i.e., slowly sinking or buoyant detrital POC escaping measurements) or the existence of yet unaccounted deep-sea (autotrophic) organic carbon supply. The latter hypothesis seems more and more plausible since it goes along with recent findings on the existence of microbial bicarbonate fixation in the dark (Reinthaler et al., 2006; Steinberg et al., 2008; Tamburini et al., 2009, 2013). The existence of a positive net community production (based on local autotrophic activities) in the deep sea, would also explain the isotopic composition of the oceanic DIC, strongly depleted in ^13^C relative to atmospheric CO_2_ (Williams et al., 2013).

Despite its recognized importance, ABD is a still poorly understood process, which is being gathering great research attention. As to the “dark ocean,” the planktonic *Thaumarchaeota* belonging to Marine Group 1 (MG1), recently defined as the candidate order *Nitrosopumilales* (Stieglmeier et al., 2014). These tiny microorganisms (usually less than 0.5 μm in size) dominate the prokaryotic cell numbers in meso- and bathypelagic realms (Karner et al., 2001; Schleper et al., 2005; Ingalls et al., 2006; Varela et al., 2008) and likely play a crucial role as nitrifying autotrophs (Herndl et al., 2005; Yakimov et al., 2007, 2011; La Cono et al., 2013, 2015; Molari et al., 2013). Globally, marine ammonium-oxidizing chemoautotrophs may fix ~0.04–0.11 Pg (×10^15^ g) of inorganic carbon annually so that making a consistent input of new organic carbon to the ocean (Herndl et al., 2005; Wuchter et al., 2006; Hügler and Sievert, 2010). Thinking in numbers, dark primary production, or ABD, represents 15–53% of the photic zone's exported production in the North Atlantic area (Reinthaler et al., 2010). Unluckily, none MG1 representatives from the meso- and bathypelagic realms of the ocean have been yet cultivated. First cultivated representative of MG1, “*Ca*. Nitrosopumilus maritimus” SCM1, has been isolated from a seawater aquarium biofilter (Könneke et al., 2005) and other closely related cultivated organisms have recently been obtained from estuarine and arctic marine sediments (Mosier et al., 2012; Park et al., 2014) and from surface coastal waters (Qin et al., 2014; Bayer et al., 2016).

In the present study we report on microbial DI^14^C fixation rates, respiration and heterotrophic production in the oxygenated meso- and bathypelagic waters (≥200 m depth) of the Mediterranean Sea. Contrary to the Mediterranean photic layers, which are characterized by an eastward steeply decreasing gradient of trophic conditions (Siokou-Frangou et al., 2010), the Mediterranean deep-sea compartments show relatively homogeneous trends of prokaryotic heterotrophic production, bulk respiration and enzymatic activities (La Ferla et al., 2010; Azzaro et al., 2012; Luna et al., 2012; Caruso et al., 2013; Celussi et al., 2017). Our work demonstrates that ABD substantially contributes to the organic carbon demand of the Mediterranean deep-sea microbial food-web so that possibly influencing meso- and bathypelagic trophic trends in the region. By the way of an empirically optimized culturing methodology, for the first time to date, we obtained highly enriched populations of thaumarchaeal deep-sea ecotype, of which activity measurements justify the importance of ABD reactions in *de novo* formation of dark-ocean organic carbon. Thus, the present data confirm previous hypotheses on the role of DIC fixation rates in the deep Mediterranean Sea (Yakimov et al., 2007, 2011, 2014; Tamburini et al., 2009; Smedile et al., 2013; Celussi et al., 2017).

## Materials and methods

**Figure 1:**
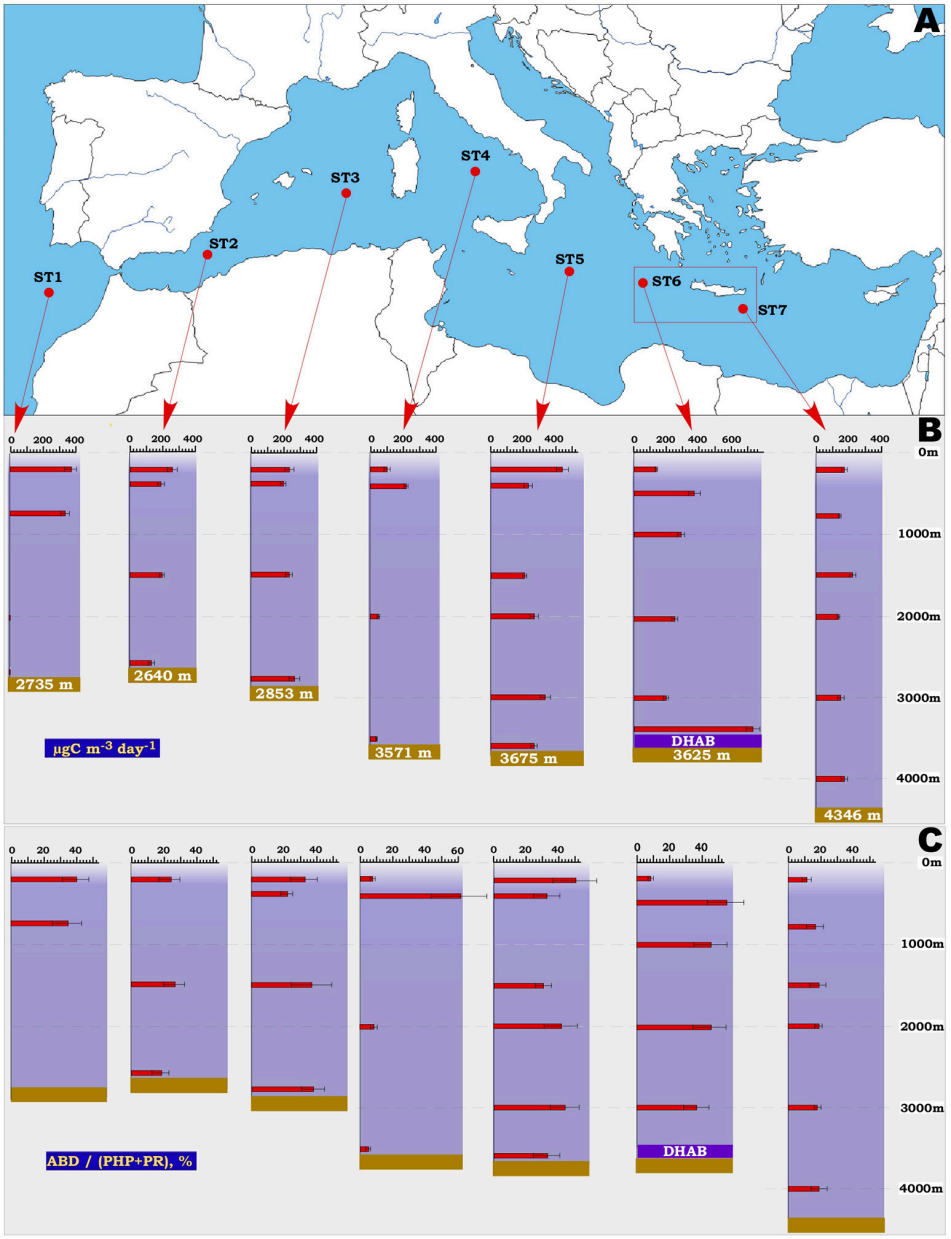
Location of the ST1-ST7 stations **(A)**, assimilation of bicarbonate in the dark (ABD) rates **(B)** and relative contribution of ABD to the total carbon demand **(C)** measured in aphotic water column along the sampling stations in the Atlantic Ocean and Mediterranean Sea. The area of the Mediterranean deep-sea hypersaline anoxic lakes (DHAB) is indicated as red rectangle. Abbreviations used: ABD, assimilation of bicarbonate in the dark; PHP, prokaryotic heterotrophic production; PR, prokaryotic respiration.

Seven hydrological stations, located from the Atlantic Ocean to the Levantine Sea (Eastern Mediterranean) were chosen as representatives of the main Mediterranean regions (Figure 1). Principal sampling of the west transects of Mediterranean basin (stations ST1-ST4) was conducted during a synoptic oceanographic cruise TRANSMED (May–June 2007) on board of Italian research vessels *Urania* and *Universitatis* in the framework of the VECTOR Line 8 research project. Three additional consecutive oceanographic cruises, MAMBA2010 (June 2010), MICRODEEP2012 (September–October 2012) and SALINE2014 (October–November 2014), were conducted to sample remaining three stations ST5-ST7 (Eastern Mediterranean) and six deep-sea hypersaline anoxic lakes (DHAL), all located in the Ionian Sea (Table 1). To measure conductivity, temperature, pressure and oxygen, a calibrated Seabird SBE9/11+ CTD was mounted on a General Oceanic's rosette sampler together with 24 Niskin bottles each of 10 L volume. Seawater samples were taken from the top of aphotic water column (200 m depth) down to the seabed (30 m above the bottom). Depths, corresponding to oxygen-minimum zone and well-defined water masses, were also sampled. All 10-L Niskin bottles were equipped with silicone rubber closure and tubing that had been carefully cleaned to avoid introducing contaminants during sampling. Besides the CTD measurements, the Winkler method (Carpenter, 1965) with an automatic burette 716 DNS Titrino (Metrohm AG, Herisau, Switzerland) was additionally carried out to measure oxygen concentration at some depths. Samples for phosphate and nitrate (20 mL) detection were directly collected from the Niskin bottles and stored at −20°C in acid-washed polyethylene vials. Nutrient concentrations were determined in triplicates within 1 month in the laboratory, using a “SEAL QuAAtro39” high performance microflow analyzer following classical methods (Grasshoff et al., 1999). Ultra cleaned glass bottles were used for ammonium measurements to avoid contamination and ${\text{NH}}_{4}^{+}$ concentration was determined fluorometrically as described elsewhere (Holmes et al., 1999; Pujo-Pay et al., 2011).

**Table 1:** The list of the cruises and sampling sites studied.

**Cruise**	**Date**	**Coordinates**	**Site name**
TRANSMED	30/05/2007	34° 59′ 59″N, 08° 19′ 58″W	ST1^a^
	01/06/2007	36° 30′ 58″N, 01° 00′ 02″W	ST2^a^
	03/06/2007	39° 19′ 18″N, 06° 04′ 47″E	ST3^a^
	08/06/2007	39° 29′ 57″N, 12° 59′ 57″E	ST4^a^
MAMBA2010	16/06/2010	33° 59′ 42″N, 26° 02′ 18″E	ST7^a^
MICRODEEP2012	19/09/2012	35° 13′ 51″N, 21° 28′ 26″E	SAL5^a^^,^ ^b^^,^ ^c^
	20/09/2012	34° 40′ 09″N, 22° 08′ 42″E	DHAL *Thetis*^b^
	23/09/2012	34° 19′ 35″N, 22° 33′ 38″E	DHAL *Medee*^b^
	25/09/2012	34° 57′ 10″N, 22° 01′ 42″E	KRY^b^^,^ ^c^
	27/09/2012	35° 18′ 19″N, 21° 23′ 21″E	ST6 (ATA)^a^^,^ ^b^^,^ ^c^
	28/09/2012	36° 29′ 34″N, 15° 39′ 34″E	ST5^a^
SALINE2014	27/10/2014	35° 16′ 37″N, 21° 41′ 22″E	DHAL *Discovery*^b^

a *Study performed on aphotic water column*.

b *Study performed on oxic/anoxic interface of the deep-sea hypersaline anoxic lakes (DHAL)*.

c *Site, used for enrichment setting (see Materials and Methods)*.

### Total prokaryotic abundance (PA), heterotrophic carbon production (HCP), prokaryotic respiration (PR) and assimilation of [^14^C]-bicarbonate at the dark (ABD)

Samples for PA, PHP, PR, and ABD were collected using 10 L Niskin bottles. Processing of the samples, lasting from water collection to incubation with the radiolabeled tracers always took less than 20 min. Epifluorescence microscopy was used for total PA determination in formalin fixed samples (2% final concentration) stained with 2,3-diamidino phenylindole (DAPI). AXIOPLAN2 Imaging microscope (Zeiss) equipped with digital camera. To obtain adequate estimates, at least 200 cells per each sample were counted and the AXIOVISION 3.1 software was used.

The PHP rates was estimated with the [^3^H]-leucine incorporation approach (Kirchman et al., 1985) using the micro-method developed by Smith and Azam (1992). Following this protocol, from each experimental sampling, five 2.0 mL vials (triplcate and two killed controls) were filled with 1.7 mL of seawater (maximal volume allowed). A total of 100 μL of cold 50% trichloroacetic acid (TCA) was used to kill controls. After 15 min, 20 nmol L^−1^ of L-[4,5-^3^H] leucine (144.2 Ci mmol^−1^; Amersham Biosciences UK Limited) was added to all samples, which were incubated in the tank for 2.5 h in the dark at *in situ* temperature. Duplicate blanks were incubated similarly to the samples, which were after incubation killed by adding cold TCA (5% final concentration). This exposure was chosen after time course experiments, performed by PHP micro-method with meso- and bathypelagic waters during two oceanographic cruises (2006-2007) in the Central-Eastern sector of Mediterranean Sea. Rates of [^3^H]-leucine incorporation increased linearly over time up to 5 h and PHP values of ≥94% of V_max_ has been observed for incubation between 120 and 150 min (Table S1). Obtained results were in consistence with the time course experiments, performed by Luna et al. (2012) with epi-, meso- and bathypelagic waters, collected at different stations across Mediterranean Sea (from 06°22′W to 26°41′E). The experimental determination of the isotopic dilution of leucine (ID) was done according to the kinetic method of Pollard and Moriarty (1984) using for each sample a 3 replicates and 2 TCA-killed controls, inoculated with a fixed concentration of ^3^H-leucine (5 nmol L^−1^) and variable concentrations of ^1^H-leucine (0, 2.5, 5, 10, 20, 40 nmol L^−1^). Our data demonstrated that 20 nmol L^−1^ of [^3^H]-leucine saturated the prokaryotic incorporation and that in meso- and bathypelagic waters the isotopic dilution values varied between 1.0 and 1.33 with a mean ID = 1.28, which provides a conversion factor of 1.92 Kg C mol^−1^ leu. The detected ID values are in accordance with those found by Van Wambeke et al. (2000) in the NE Mediterranean Sea and were used through our study in spite of the theoretical conversion factors with or without ID, previously applied for meso- and bathypelagic waters of the Mediterranean Sea (Luna et al., 2012; Celussi et al., 2017). After extraction of ^3^H-labeled proteins (Smith and Azam, 1992), activity in the samples was determined in a Wallac 1414 liquid scintillation counter (PerkinElmer, Monza, Italy). The instrument was calibrated with internal and external standards. The blank-corrected leucine incorporation rates were converted into PHP values using the experimentally estimated ID values.

The Electron Transport System (ETS) assay based on the technique of the tetrazolium reduction (Packard et al., 1988) was used to estimate potential respiratory activity rates of the entire microbial community. Some minor modifications of the method were made to increase its sensitivity (La Ferla et al., 2003, 2005; Azzaro et al., 2006). Briefly, to remove large particles, subsamples for the analysis (10–20 L) were pre-filtered on a 200 μm pore size mesh net and then filtered through a Whatman GF/F glass fiber membranes (0.5 μm pore size; 47 mm diameter) at reduced pressure (≤0.3 bar). Filters were placed into 15 mL Falcon tubes and immediately stored in liquid nitrogen until analysis in the laboratory (≤45 days). The ETS data were corrected for *in situ* temperature with the Arrhenius equation using a value for the activation energy of 15.8 kcal/mol (Packard and Williams, 1981; Packard et al., 1988; Reinthaler et al., 2006). Obtained ETS assay values were further converted into PR rates applying the Takahashi oxygen to carbon molar ratio (Takahashi et al., 1985) and conversion PR: ETS factor of 0.68 (measurements made in mesopelagic layers of the subtropical Atlantic; Arístegui et al., 2005). Compared to other conversion factors, estimated either in nutrient-rich batch culture (Christensen et al., 1980) or in cold meso- and bathypelagic layers of North Atlantic (Reinthaler et al., 2006), the above PR/ETS ratio produced values of the prokaryotic growth efficiency (PGE = PHP/[PHP+PR]) in a range of 0.3–16%, coinciding with PGE values obtained by performing *in situ* marine studies (Del Giorgio and Cole, 1998 and references therein; Reinthaler et al., 2006, 2010; Azzaro et al., 2012; Celussi et al., 2017).

For ABD incubations, 40 mL of alive samples and formaldehyde-fixed blank controls were placed in 60 mL gas-tight serum bottles. The ABD rates were estimated by addition of 10 μCi of [^14^C]-bicarbonate (56.0 mCi mmol^−1^, Amersham Italia, Milan, Italy) using a gas-tight Hamilton syringe to yield a final activity of 0.25 μCi mL^−1^. Triplicate samples and duplicate formaldehyde-killed blanks were incubated in the dark at 14°C for 72 h and then stopped by the addition of formaldehyde (2% final concentration). Previously, we confirmed the linearity of [^14^C]-bicarbonate incorporation in microbial biomass (y = 0.42857 + 4.3393x ([R = 0.99354]) during at least 72 h of incubation time without significant changes in cell concentrations (Yakimov et al., 2014). All samples were thereafter filtered through 0.1 μm polycarbonate filters (Millipore) and rinsed three times with 10 mL of ultra-filtered seawater. Subsequently, before addition of scintillation cocktail for [^14^C]-counting, the washed filters were acidified for 12 h in an HCl fume hood to remove inorganic carbon and air-dried. Filters were than stored at −20°C until the radioactivity was counted in a Wallac 1414 analyser (Perkin Elmer, Monza, Italy). According to protocols of dark DIC fixation measurements (Herndl et al., 2005; Reinthaler et al., 2010), the disintegration per minute (DPM) values was calculated by subtracting the values detected in the abiotic controls from the absolute DPM obtained in the samples. Integrated ABD values (mg C m^−2^ day^−1^) were calculated by means of the trapezoidal method (Moutin and Raimbault, 2002) using the discrete data and assuming that rates at the bottom were identical to those of the deepest sampled depth.

The Kolmogorov-Smirnov test was used for assessing normality of datasets. To determine the significance of measured rates, One-Way or were specified Two-Ways ANOVA analysis of variance was applied. Relative importance of each treatment group was investigated by pairwise Multiple Comparisons procedures (Holm-Sidak method) with overall significance level 0.05. As described elsewhere (La Cono et al., 2013), calculations were carried out using SigmaStat software for Windows, ver. 3.1 (Copyright 1992–1995; Jandel Corporation) and differences were considered significant at *P* < 0.05.

### Establishment of enrichment cultures

Seawater samples (~1 L of volume) were collected during MICRODEEP2012 cruise from the upper interface of the brine lakes *L'Atalante* (3,499 m depth) and *Kryos* (3,338 m depth) referred to as the ATA and KRY samples, respectively. Additionally, one bathypelagic sample (3,000 m depth) was collected during the same cruise over the brine lake *Urania* (35°13′51′′N; 21°28′24′′E; 3,552 m depth) and referred to as the SAL5 sample (Table 1). At time of sampling, ammonium concentrations varied between 0.48 μM (SAL5) and 120–450 μM (ATA and KRY). Enrichment cultures were initiated by filtering of 900 mL seawater through 0.45 μm polycarbonate filters (Millipore) and addition of 100 mL autoclaved bathypelagic seawater supplemented with KH_2_PO_4_ (1 mM), NaHCO_3_ (10 mM), Fe-NaEDTA (20 μM), non-chelated trace elements (10×), selenite-tungstate (10×) and vitamins (10×) solutions (Widdel and Bak, 1992). The pH of the medium was stabilized at 7.8 by adding of HEPES buffer (1 M HEPES, 0.6 M NaOH). In concordance with elevated ambient concentrations of ammonia in the DHAL interfaces, 500 μM NH_4_Cl was added to ATA and KRY cultures. In contrast, the ammonia-impoverished bathypelagic sample SAL5 was initially supplemented with 100 μM NH_4_Cl. Finally, all enrichments were supplemented with 100 μM α-ketoglutarate to support eventual mixotrophic requirements of isolates (Qin et al., 2014). Cultures were incubated in the dark at 16°C without shaking. Applying the absorbance spectroscopy method (Strickland and Parsons, 1972), ammonia-oxidizing activity in the enrichment cultures was monitored by nitrite production. Additionally, microbial growth was monitored by DAPI (Glöckner et al., 1999). After reaching a plateau in nitrification, 10% (vol/vol) of the total culture volume was transferred to a fresh medium and cultivated under the same conditions. Unless otherwise stated, the enrichment cultures ATA and KRY were supplemented with 500 μM ammonia and SAL5 with 100 μM ammonia as energy source. The pH of the medium remained almost constant (7.6–7.8) during the culture cycle. No antibiotics were added to avoid loss of eventually antibiotic-sensitive deep-sea ecotypes.

### Gene cloning, sequencing, and phylogenetic analyses

Prokaryotic 16S *rRNA* and thaumarchaeal key genes involved in ammonia respiration (ammonia monooxygenase, *amo*A) and autotrophy (4-hydroxybutyryl-CoA dehydratase, *hbd*), were amplified by PCR using the primers listed in La Cono et al. (2010, 2013). All reactions were carried out in a MasterCycler 5331 Gradient PCR (Eppendorf, Hamburg, Germany) under the conditions for PCR as described elsewhere (La Cono et al., 2010, 2013; Yakimov et al., 2013). The PCR products were further purified PCR purification QIAQuick column (Qiagen, Germany) and cloned into pGEM T-Easy Vector II. After PCR verification, positive clones from each library were sequenced at Macrogen (Amsterdam, Netherlands). Pintail software (Ashelford et al., 2006) and CHECK_CHIMERA (available from Ribosomal Database Project) were used to check sequences for possible chimeric origin. After manual checking, phylogenetic trees of the 16S rRNA gene amplified sequences and close relatives identified with BLAST (Altschul et al., 1997) were created using the ARB and SILVA alignment tools (Ludwig et al., 2004; Pruesse et al., 2007). MEGA 5 (Tamura et al., 2011) and MacVector 11.1.2 were used to align sequences of *amo*A and *hbd* functional genes. After alignment, the neighbor-joining algorithm of ARB and MEGA 5 program packages were used to generate the phylogenetic trees based on distance analysis for 16S rRNA and functional genes, respectively. To estimate the robustness of inferred topologies and the reproducibility of the trees, 1,000 bootstrap re-sampling was tested using the same distance model.

### Nucleotide sequence accession numbers

The nucleotide sequences have been submitted in the DDBJ/EMBL/GenBank databases under accession numbers: MF624634 to MF624712 for the archaeal and bacterial 16S rRNA gene sequences, MF662830 to MF662891 for the thaumarchaeal *amo*A gene sequence, MF662892 to MF662967 for the thaumarchaeal *hbd* gene sequences.

## Results and discussion

### Latitudinal and vertical trends of prokaryotic parameters in the deep mediterranean sea

Spatial patterns of relevant physical variables, as well as prokaryotic parameters (PHP, PR, PGE) and contribution of ABD to prokaryotic carbon demand, measured along the longitudinal gradient of the Mediterranean Sea, are reported in Table 2 as the mean values and the range of standard deviations. Hydrographical data revealed that the Atlantic station (ST1) was characterized by colder and lower salinity water masses. As a consequence, all prokaryotic parameters measured at ST1 below 750 m were among the lowest (Table 2). When considering the seven stations sampled, the total prokaryotic abundance decreased significantly with depth (*p* < 0.001). Moreover, as the second commonly reported pattern, the prokaryotic abundance was observed be slightly higher in the Algero-Balear sub-basin, compared with the rest of the deep Mediterranean Sea (Two Way ANOVA *p* = 0.005 or *p* < 0.001 using geographic location or depth as a factor, respectively). Prokaryotic respiration rates measured with the ETS method did exhibit evident downward-decreasing trend (*p* < 0.05) although they were apparently uniform in both Mediterranean Sea basins. The prokaryotic heterotrophic production (PHP) showed the fastest rates in the eastern basin (ST7) with the maximum values, detected in the deepest layers (125.3 ± 4.5 and 97.7 ± 7.4 μg C m^−3^ d^−1^ at depth of 3,000 m and 4,000 m, respectively). Noteworthy, the PHP rates measured in bathypelagic water masses at majority of locations (ST2-5 and ST7) were significantly higher (*p* < 0.001) than in corresponding mesopelagic compartments (Table 2). The highest difference was observed at station ST3, where the heterotrophic production measured at depth of 2,837 m was more than three times higher (97.4 ± 1.9 μg C m^−3^ d^−1^) than uppermost PHP values (28.6 ± 0.6 μg C m^−3^ d^−1^). Our calculated PGE values (0.3–16%) were similar to the estimates previously calculated for similar areas (Azzaro et al., 2012; Celussi et al., 2017), substantiating the correctness of the conversion factors used in present study. Thus, taken together all aforementioned measurements, which are coincidental with previously reported data (La Ferla et al., 2010; Zaccone et al., 2010, 2012; Luna et al., 2012; Caruso et al., 2013; Celussi et al., 2017), we found that the deep Mediterranean Sea did possess neither the eastward- nor downward-decreasing gradient of main prokaryotic parameters, characteristic for sunlit layers of the Mediterranean Sea (Sarmiento et al., 1988; Danovaro et al., 1999; Thingstad et al., 2005; López-Sandoval et al., 2011).

**Table 2:** Average water masses properties of selected physicochemical and biological parameters in the western. central and eastern Mediterranean sub-basins.

**Variables (m)**	**Sali-nity**	**Temp, (°C)**	**PHP^a^**	***SD*, *n* = 3**	**PR^a^**	***SD*, *n* = 2**	**ABD^a^**	***SD*, *n* = 3**
**STATION ST1, BOTTOM 2,735 m**								
200	36.01	14.13	19.4	0.8	967	51	394	39
750	36.06	11.69	11.0	0.4	987	45	347	27
2,000	35.17	4.58	7.7	0.0	377	19	0.40	0.01
2,728	34.97	3.10	5.5	0.0	204	8	4.4	0.4
**STATION ST2, BOTTOM 2,640 m**								
200	38.41	13.18	34.6	1.1	1,079	54	268	33
400	38.53	13.24	ND	ND	794	35	198	24
1,500	38.47	13.08	12.5	0.3	763	39	204	26
2,633	38.48	13.27	38.9	0.7	733	22	142	16
**STATION ST3, BOTTOM 2,853 m**								
200	38.30	13.52	28.6	0.6	763	28	250	20
400	38.60	13.56	8.2	0.6	946	46	210	11
1,500	38.47	13.08	13.7	0.1	631	31	240	14
2,837	38.49	13.31	97.4	1.9	651	17	281	18
**STATION ST4, BOTTOM 3,571 m**								
200	38.64	14.04	5.5	0.1	1,577	99	114	14
400	38.74	14.05	3.8	0.1	366	13	231	21
2,500	38.52	13.43	5.5	0.1	753	37	59	9
3,500	38.50	13.56	27.8	0.5	926	39	48	6
**STATION ST5, BOTTOM 3,675 m**								
200	38.94	14.81	23.0	0.2	794	41	411	31
400	38.84	14.13	7.0	0.1	662	34	215	27
1,500	38.75	13.79	5.5	0.1	662	17	200	11
2,000	38.74	13.80	64.6	2.8	550	21	249	16
3,000	38.74	13.91	44.4	1.9	672	17	312	31
3,655	38.74	13.94	37.0	3.0	712	26	248	21
**STATION ST6, BOTTOM 3,625 m**								
200	38.92	14.77	12.7	0.7	1,761	75	140	16
500	38.85	14.13	13.0	0.5	662	16	370	36
1,000	38.75	13.74	4.8	0.1	641	32	290	20
2,000	38.75	13.84	8.9	0.2	539	19	245	14
3,000	38.74	13.94	6.7	0.1	570	13	207	19
3,400	38.74	13.94	ND	ND	ND	ND	730^b^	41^b^
**STATION ST7, BOTTOM 4,346 m**								
200	39.17	17.80	89.0	4.1	1,333	69	160	21
750	38.89	14.36	33.1	0.8	814	42	133	14
1,500	38.77	13.88	34.3	0.9	1048	41	207	16
2,000	38.76	13.85	81.8	4.1	590	18	126	10
3,000	38.77	14.08	125.3	4.5	662	16	143	6
4,000	38.76	14.20	97.7	7.4	773	35	163	11

a *Values of prokaryotic heterotrophic production (PHP), prokaryotic respiration (PR) and assimilation of bicarbonate in the dark (ABD) are given in μg C m^−3^ d^−1^*.

b *Data from Yakimov et al. (2007)*.

*ND, not determined*.

### Spatial patterns of ABD in the deep mediterranean sea

Using several molecular and cultivation techniques (Herndl et al., 2005; Kirchman et al., 2007; Grote et al., 2008; Varela et al., 2011; Yakimov et al., 2014), it is now confirmed that DIC fixation is widespread among prokaryotic organisms in meso- and bathypelagic oceanic water column, including the Mediterranean Sea. It is important to point out that methodology applied through our [^14^C]-bicarbonate assimilation experiments implied to measure only the [^14^C]-incorporation into biomass remained after filtration, i.e., to measure rates of the particulate ABD. Thus, the eventual formation of the dissolved radiolabeled compounds, like released organic molecules, extracellular vesicles, signaling devices and enzymes or cellular constituents released after viral lyses (Celussi et al., 2017), was overlooked in present study. Nevertheless, our ABD data were coherent with PHP values, since the last measurement was also ignoring the formation of [^3^H]-radiolabeled compounds and cellular constituents, not settled during micro-centrifugation. Deep Mediterranean is very unique basin in a variety of ecological settings. Besides warm temperature and oligotrophy, the concentration of DIC in Mediterranean is very high and ranges from 2.19 to 2.47 mmol l^−1^ (Hassoun et al., 2015), compared to 1.72 mmol l^−1^ in reference composition of oceanic water with salinity of 35.1‰. Such a high concentration of ambient cold [^12^C]-bicarbonate obviously affect the specific uptake of added isotopic hot [^14^C]-bicarbonate (4.464 μmol L^−1^ of this isotope is typically used for DIC fixation experiments). Compared to the isotopic dilution of hot [^3^H]-leucine, used throughout present study (mean ID = 1.28), the ID values of added [^14^C]-bicarbonate are in range between 490.6 and 553.3. Thus, to obtain reliable results of ABD, we followed the common practice (Herndl et al., 2005; Reinthaler et al., 2010; Celussi et al., 2017) and increased the volume of sample and prolonged the incubation time with hot [^14^C]-bicarbonate. However, these different incubation times in PHP and ABD measurements did not pose a problem since it has been shown that leucine uptake rates as well as the bicarbonate assimilation in deep waters are linear over a period of at least 72 h (Reinthaler et al., 2006, 2010; Yakimov et al., 2014).

The westernmost Atlantic station ST1, taken for comparative reasons, was characterized by cold waters below 750 m (4.6 and 3.1°C at 2,000 m and 2,728 m depth, respectively). As a consequence, the ABD values, representing the *dark ocean primary production* rates (term coined by Herndl et al., 2005), decreased from 350 to 400 μg C m^−3^ d^−1^ at 200–750 m depths to 0.4–4.4 μgC m^−3^ d^−1^ at ST1 bathypelagic levels (Figure 1 and Table 2). These rates corresponded well to the DIC fixation rates reported for the deep North Atlantic's interior (120 and 1.0 μg C m^−3^ d^−1^ for the corresponding depths) (Reinthaler et al., 2010). The ABD values measured in dark Mediterranean Sea, unlike those of the Atlantic station ST1, did not evidently exhibit either latitudinal or vertical spatial gradients on average (Two Way ANOVA *p* = 0.629 or *p* = 0.541 using depth or geographic location as a factor, respectively; see Figure 1). Station ST4 (Tyrrhenian Sea), represented the single exception, with ABD rates dropping from 231 ± 21 to 48 ± 6 μg C m^−3^ d^−1^, (*p* = 0.006) detected in meso- and bathypelagic waters, respectively. As we discussed it already (Yakimov et al., 2011), earlier estimates of bathypelagic ABD activity (Tamburini et al., 2009) measured in this area at the depth of 3,000 m (72.0 ± 8.9 μg C m^−3^ d^−1^) coincided with our values and seem to be a characteristic feature of Tyrrhenian Deep Waters, which are the oldest, highly oligotrophic and densest deep water masses of the Mediterranean Sea (Millot et al., 2006).

In general, observed uniformity of microbial ABD rates over the 2,500-km-long transect is likely supported by the relative homogeneity of environmental parameters, fundamental for DIC fixation. Indeed, as it reported elsewhere (Danovaro et al., 1999; Yakimov et al., 2011; Santinelli et al., 2015; Celussi et al., 2017 and references herein), the whole Mediterranean interior, from roughly 300–500 m to the seabed, possesses basin-scale minor variations in salinity (<4%), ammonia (<8%), oxygen (<24%), bicarbonate concentrations (<15%), DOC (<10%) and, especially, temperature (<12%). Thus, such evenly distribution of basic environmental settings defines the deep Mediterranean ecosystem as relatively homogenous primarily-producing environment. Documented “hotspots” of autotrophic activity in deep Mediterranean take place at very specific areas, such as deep anoxic hypersaline lakes of Ionian Sea, the large hydrological formations, characterized by the existence of energetically-rich redoxclines (Yakimov et al., 2007, 2013, 2015).

### ABD rates at the surficial layers of deep hypersaline anoxic lakes

**Figure 2:**
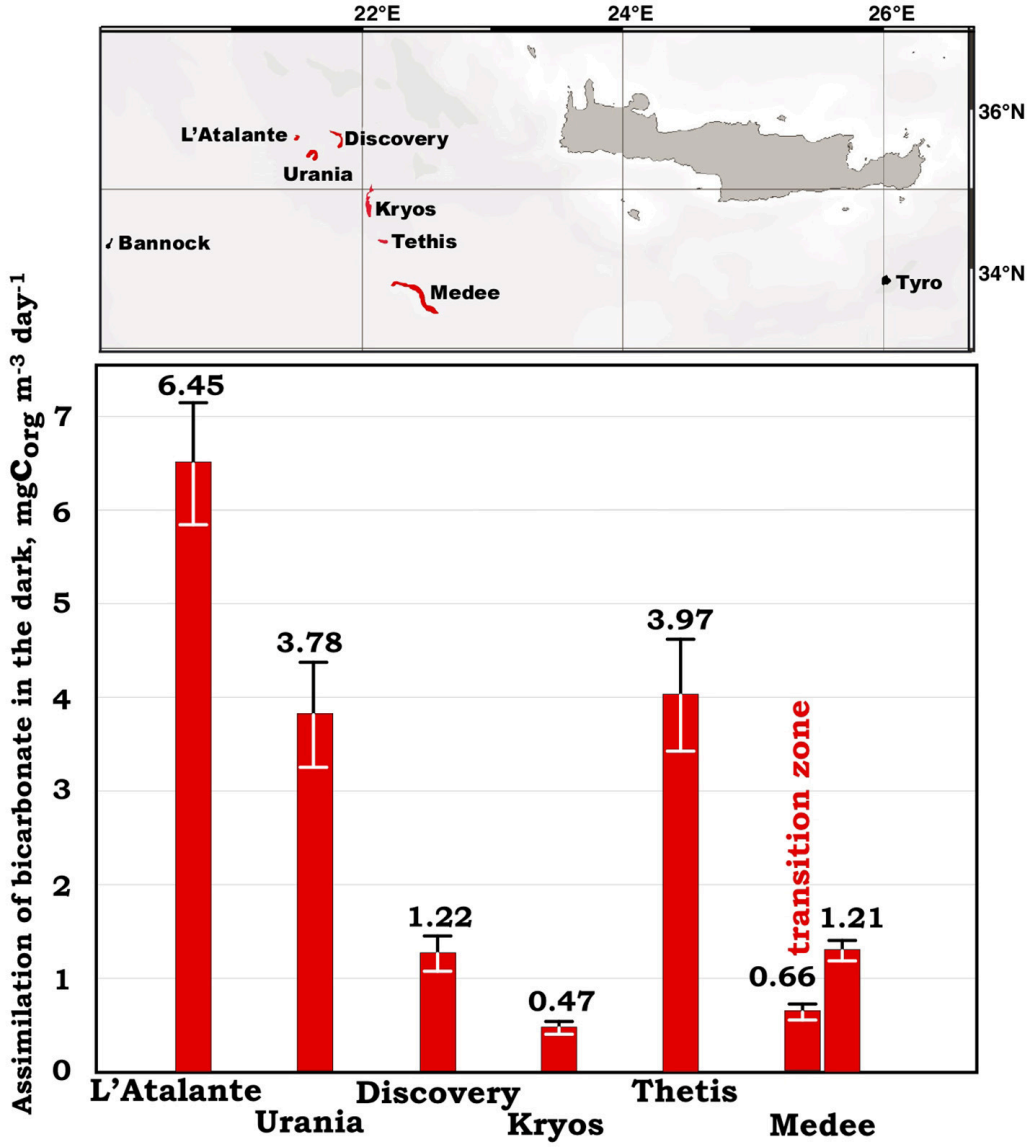
Location of all currently known Mediterraneran DHAL and dark primary production (ABD) rates, measured in oxic/anoxic surficial layer of these deep-sea lakes.

During MICRODEEP2012 and SALINE2014 cruises we measured the ABD values at the interfaces of the deep-sea anoxic hypersaline lakes *Discovery, Kryos*, and *Urania*. In our previous studies we demonstrated that these interfaces represent gradients of physical and chemical factors, which constitute major forces shaping active chemoautotrophic ecosystems (Yakimov et al., 2015 and reference therein). Indeed, very fast bicarbonate assimilation rate (3.78 ± 0.57 mg C m^−3^ d^−1^) has been detected at the upper layer of the NaCl-saturated DHAL *Urania* (Figure 2), comparable with our previous measurements performed on the other thalassohaline DHAL, *L'Atalante* and *Thetis* (Yakimov et al., 2007; La Cono et al., 2011). The lake *Medee*, while exhibiting relatively low ABD rates in the interface (1.21 ± 0.11 mg C m^−3^ d^−1^), singularly possesses a very thick and active transition zone (~40 m, 0.66 ± 0.06 mg C m^−3^ d^−1^). The DHAL *Discovery* and *Kryos* are saturated with MgCl_2_ (~5M) and represent the exceptionally chaotropic system with the lowest water activity value registered for any hydrological formation on our planet (Hallsworth et al., 2007; Yakimov et al., 2015). Due to this fact, the ABD values in the *Discovery* and *Kryos* interfaces are significantly lower, compared to that of the thalassohaline DHAL. But these rates are still considerably higher the mean value of DIC fixation rates, measured in the deep Ionian Sea. Using the ABD values calculated for the lake *Medee*, which spans over ~100 km^2^ (Yakimov et al., 2013), net CO_2_ assimilation activities were estimated as 27.4 ± 2.5 tons daily. Hence, the deep brine lakes represent the hotspot of both metabolic activity and microbial diversity and contribute to the global CO_2_ sink in the deep Ionian Sea.

### Importance of ABD in the mediterranean interior

According to the literature, the Mediterranean Sea is characterized by clear longitudinal patterns as to the “solar radiation related features.” In particular, together with a deepening of the photic zone lower limit (from 70 to 110 m below the sea surface), it shows decreasing trends of phototrophic primary productivity (PhPP) eastward (Moutin and Raimbault, 2002; Siokou-Frangou et al., 2010; López-Sandoval et al., 2011). In order to compare ABD with phototrophic primary production along the sampled longitudinal transect, we uniformed and averaged the PhPP values, estimated *in situ* and originally obtained from the literature (Moutin and Raimbault, 2002; Siokou-Frangou et al., 2010), for each area, corresponded to Mediterranean stations ST2-ST7 (Table 3). Additionally to these data, the integrated net PhPP values, deduced from the 3-D-biogeochemical OPATM-BFM modeling approach (Lazzari et al., 2012), were also taken into consideration. Depth-integrated ABD assimilation rates in the Mediterranean deep waters ranged from 396 ± 49 (station ST4) to 873 ± 99 mg C m^−2^ d^−1^ (Table 3). Unlike the west-east decreasing trend in the integrated net phytoplankton primary production, the ABD rates showed centripetal increase trends, with peaks of ABD activity observed in the central stations ST5 and ST6 (mean value for ST5+ST6: 867 mg C m^−2^ d^−1^). This may result from at least two reasons. Although the ST2 and ST3 stations of the Western Mediterranean possess the ABD activity (mean value is 224 ± 25 μg C m^−3^ d^−1^), similar to that of the central stations ST5 and ST6 (mean value is 262 ± 33 μg C m^−3^ d^−1^), this region is considerably shallower. Secondly, the effects of the nearby processes of dense water formation with participation of nutrients-rich Adriatic waters (Korlević et al., 2015) and ammonium- and reductants-rich DHAL most likely define elevated ABD activities, compared to extremely oligotrophic Levantine sub-basin. In conclusion, the measured ABD rates in the Mediterranean Sea accounts for 85–424% of the phototrophic primary production (Table 3), thus clearly highlighting the significance of dark DIC fixation to support meso- and bathypelagic carbon demand. Indeed, the mean ratio of ABD: prokaryotic carbon demand (PCD = PHP + PR) ranged from 5 to 62% (Figure 1 and Table 2). Thus, along the latitudinal transect, most of deep Mediterranean aphotic waters exhibited capacity to self-compensate their PCD on 20–50% by *de novo* production organic carbon in the dark.

**Table 3:** Regional averages of integrated net phototrophic primary production (PhPP), reported for Mediterranean Sea, and ABD rates, estimated in present study.

**Sub-basin**	**Net PhPP rates (mg C m**^**−2**^ **d**^**−1**^**)**		**Net ABD rates (mg C m^−2^ d^−1^)^c^**
	***In situ*^14^C^a^**	**OPATM-BFM^b^ model**	
Alboran Sea	448 ± 188	545 ± 321	465 ± 71 (ST2)
Catalan Balearic	533 ± 367	509 ± 199	645 ± 48 (ST3)
Tyrrenian Sea	367 ± 83	279 ± 118	396 ± 49 (ST4)
Ionian Sea	324 ± 126	189 ± 99	873 ± 99 (ST5)
			864 ± 89 (ST6)
Levantine	149 ± 80	208 ± 110	632 ± 61 (ST7)

a *Data from Siokou-Frangou et al. (2010)*.

b *Data from Lazzari et al. (2012)*.

c *Average of integrated ABD rates at the ST2-ST7 stations, estimated in present study*.

Although it was not the objective of the present study, we are aware that decompression of the samples retrieved from greater depth might influence prokaryotic activities, measured at ambient pressure. Since we obtained even distribution of both prokaryotic growth yields and ABD rates in meso- and bathypelagic waters, this would indicate that both production/respiration and assimilation of bicarbonate responded similarly. Whether decompression stimulates or affects the biological activities of deep-sea prokaryotes is still unclear and contradictory (Tamburini et al., 2013 and references therein). Indeed, stimulation of deep water prokaryotic activity was reported in some studies (Jannasch and Wirsen, 1982), while other authors detected inhibition of activity because of decompression (Tamburini et al., 2003, 2009). Thus, lack of general consensus about the pressurized vs. decompressed metabolic activities of deep-sea microbiota yet has to be resolved. Despite of these and other uncertainties, related to the conversion factors and activity calculations, the deep Mediterranean Sea seems significantly contribute to *de novo* production of organic matter and to the global carbon cycling in the basin. This is likely the result of combination of several unique characteristics of Mediterranean Sea, such as high abyssal temperature and the ultra-oligotrophy of the Central-Eastern region (Thingstad et al., 2005).

### Cell-specific prokaryotic carbon demand and ABD rates

Over the 2,500-km-long transect, the bicarbonate assimilation rates measured in all our samples, apart of the very active samples collected over the DHAL, ranged from 48 ± 6 to 411 ± 31 μg C m^−3^ d^−1^ (Figure 1 and Table 2). These data coincided with our previous measurements, performed in the Tyrrhenian and Ionian Sea (Yakimov et al., 2007, 2011, 2014; Smedile et al., 2013). Besides these measurements, there are very few data available for dark DIC fixation in deep Mediterranean waters. They demonstrated remarkable discrepancy spanning from 50 to 7,350 μg C m^−3^ d^−1^ (Tamburini et al., 2009; Celussi et al., 2017). The fastest ABD uptake rates, measured by Celussi et al. (2017) in the Algero-Balear and Levantine sub-basins (7.35 and 3.85 mg C m^−3^ d^−1^, respectively) are only comparable to our data measured in the upper horizon of DHAL (Figure 2). It is important to remark, as the elevated concentrations of reduced compounds (mainly hydrogen sulfide), originated from lake's anoxic interior, can likely fuel such a high activity of dark DIC fixation in the DHAL interfaces. In contrary, the energy sources for CO_2_-assimilating microbes in the oxygenated aphotic water column are scanty and hardly sufficient to support the same rates of primary production. Elucidating this question, we analyzed in more details the cell-specific prokaryotic carbon demand (csPCD) and the cell-specific assimilation of bicarbonate in the dark (csABD).

Although prokaryotic abundance decreased exponentially with the depth, both csPCD and csABD showed opposite changes and the cell-specific values significantly increased toward the deeper water masses (*r* = 0.958, *p* < 0.04 for the western and *r* = 0.935, *p* < 0.001 for the eastern sub-basins). With only one exception of cold deep Atlantic waters (ST1), the highest csPCD and csABD rates were measured at the greatest depths (Table 4). In all studied stations of the Mediterranean basin, the cell-specific PCD values ranged from 1.72 ± 0.15 fg C cell^−1^ d^−1^ (200 m depth, station ST3) to 19.87 ± 2.48 fg C cell^−1^ d^−1^ (3,500 m depth, station ST4). These results are similar to previous oceanographic studies, where the cell-specific PCD values of 8–36 fg C cell^−1^ d^−1^ were reported (Reinthaler et al., 2006). Compared to the csPCD, the cell-specific ABD rates were more homogenous in whole aphotic Mediterranean and varied between 0.38 ± 0.08 fg C cell^−1^ d^−1^ (200 m depth, station ST7) and 3.91 ± 0.57 fg C cell^−1^ d^−1^ (3,500 m depth, station ST6). Taking into account an average of carbon content per cell (10–20 fg C cell^−1^) (Danovaro et al., 2008; Kallmeyer et al., 2012), this assimilation activity could likely support the autotrophic microbial growth with a generation time of 2.5–50 days. As we mentioned above, the planktonic *Thaumarchaeota* belonging to MG1 dominate the prokaryotic cell numbers in ocean interior and seem play an important role in DIC fixation in the aphotic meso- and bathypelagic ocean (Francis et al., 2005; Herndl et al., 2005; Reinthaler et al., 2010; Middelburg, 2011; Tolar et al., 2016). Cells of the first cultured representative, “*Candidatus* Nitrosopumilus maritimus” SCM1, are capable to grow autotrophically to a density of 3 × 10^7^ cells·mL^−1^ (~0.6 μg mL^−1^ dry mass) with a generation time of 35 h, which requires a carbon-fixation rate of 39 nmol·d^−1^·μg^−1^ protein (Könneke et al., 2014). As the average of crude protein content in microbial biomass is 40–60% dry weight, these values corresponded to cell-specific fixation rates of 3.7–5.6 fg C cell^−1^ d^−1^. Overall, statistical summaries of our csABD data corresponded well with the literature values of microbial cell-specific DIC assimilation, including the data of cultivation trials (Lenk et al., 2011; Swan et al., 2011; Könneke et al., 2014; Yakimov et al., 2014; Dyksma et al., 2016; Cao et al., 2017).

**Table 4:** Prokaryotic abundance (PA), cell-specific prokaryotic carbon demand (csPCD) and cell-specific assimilation of bicarbonate in the dark (csABD) in the water masses of the Mediterranean sub-basins.

**Variables (m)**	**PA, 10^3^ cell mL^−1^**	**SD**	**csPCD, fg C cell^−1^ d^−1^**	***SD***	**csABD, fg C cell^−1^ d^−1^**	***SD***
**STATION ST1. BOTTOM 2,735 m**						
200	240	21	4.11	0.57	1.64	0.39
750	180	8	5.54	0.49	1.93	0.24
2,000	92	8	4.18	0.57	0.004	0.001
2,728	75	7	2.79	0.36	0.06	0.01
**STATION ST2. BOTTOM 2,640 m**						
200	450	42	2.47	0.35	0.60	0.13
400	350	27	ND	ND	0.57	0.12
1,500	260	19	2.98	0.37	0.78	0.16
2,633	170	14	4.54	0.35	0.84	0.16
**STATION ST3. BOTTOM 2,853 m**						
200	460	24	1.72	0.15	0.54	0.07
400	150	9	6.36	0.69	1.40	0.16
1,500	220	11	2.93	0.29	1.09	0.11
2,837	180	8	4.16	0.29	1.56	0.17
**STATION ST4. BOTTOM 3571 m**						
200	187	19	8.46	1.39	0.61	0.14
400	131	9	2.82	0.29	1.76	0.28
2,500	99	7	7.66	0.91	0.60	0.13
3,500	48	4	19.87	2.48	1.00	0.21
**STATION ST5. BOTTOM 3675 m**						
200	170	13	4.81	0.60	2.42	0.37
400	132	11	5.07	0.68	1.63	0.34
1,500	122	11	5.47	0.63	1.64	0.24
2,000	72	6	8.54	1.04	3.46	0.51
3,000	84	6	8.53	0.83	3.71	0.63
3,655	79	9	9.48	1.44	3.14	0.62
**STATION ST6. BOTTOM 3625 m**						
200	221	17	8.03	0.96	0.63	0.12
500	140	8	4.82	0.39	2.64	0.41
1,000	150	12	4.31	0.56	1.93	0.29
2,000	75	5	7.31	0.74	3.27	0.40
3,000	53	3	10.88	0.86	3.91	0.57
3,400	1005^a^	65^a^	ND	ND	0.73	0.09
**STATION ST7. BOTTOM 4346 m**						
200	423	34	3.36	0.44	0.38	0.08
750	218	13	3.89	0.42	0.61	0.1
1,500	205	8	5.28	0.41	1.01	0.12
2,000	127	9	5.29	0.54	0.99	0.15
3,000	136	16	5.79	0.83	1.05	0.17
4,000	84	8	10.37	1.50	1.94	0.31

a *Data from Yakimov et al. (2007)*.

*ND, not determined*.

However, it seems that ammonium-oxidizing *Thaumarchaea* are not the only player of ABD in the deep Mediterranean (Yakimov et al., 2014 and references herein). Relying on availability of other electron donors (i.e., reduced sulfur intermediates, CO and H_2_), chemolithoauto- and chemoorganotrophic communities, primarily sulfur-oxidizing and carboxydotrophic bacteria, are capable to perform assimilation of bicarbonate (Swan et al., 2011; Anantharaman et al., 2013). Additionally, a vast majority of heterotrophic bacteria can incorporate CO_2_ using metabolic pathways not necessary related to autotrophy. Depending on organic compounds for carbon supply, these heterotrophic organisms fix carbon dioxide via a variety of carboxylation reactions, including those of anaplerosis (Romanenko, 1964; Hesselsoe et al., 2005, 2008; Alonso-Sáez et al., 2010; Llirós et al., 2011; DeLorenzo et al., 2012; Yakimov et al., 2014). The autotrophic potential in marine mesophilic bacterial isolates is consistent with the CO_2_ assimilation activity of “*Candidatus* Nitrosopumilus maritimus” SCM1 and can reach the levels of 0.5–7.5 fg C cell^−1^ d^−1^ depending on bacterial isolates (Lenk et al., 2011; Yakimov et al., 2014; Dyksma et al., 2016; Cao et al., 2017).

Unlike both these literature values and our calculations, Celussi et al. (2017) reported very high estimates of the MS dark primary production data. Combining estimates of total prokaryotic abundance at bathypelagic (>2,000 m) depths with these ABD data, we assessed that the cell-specific CO_2_ fixation rates in this case have to be very high, namely 24–35 fg C cell^−1^ d^−1^ for bathypelagic water masses of Agero-Balear and Ionian stations and even higher, up to 70 fg C cell^−1^ d^−1^, for bathypelagic water masses of Tyrrhenian Sea. Such cell-specific ABD values are considerably different from our data and from those obtained by culturing of CO_2_-fixing isolates in optimized media. At the moment we are at a loss to explain such discrepancy. Obviously, more studies are needed both to unify the measurements of the deep-water DIC fixation rates and to cultivate the key players of this process with the subsequent studies on their ecophysiology.

### Establishment of actively nitrifying and CO_2_-fixing enrichment cultures

**Figure 3:**
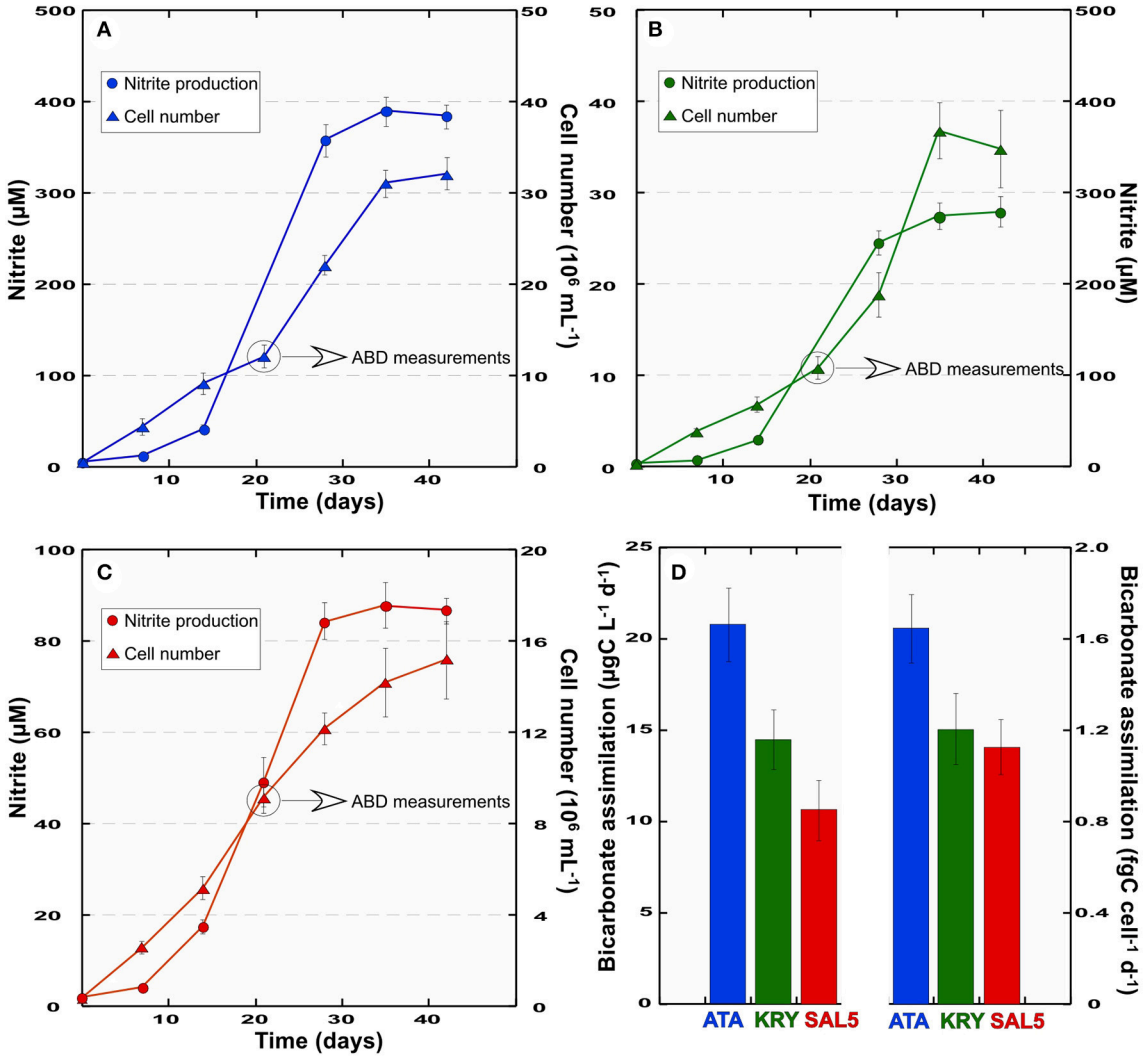
Growth curves (triangles) and nitrite production (circles) of ATA **(A)**, KRY **(B)**, SAL5 **(C)** enrichments and their total and cell-specific bicarbonate assimilation rates, ABD and csABD, respectively **(D)**. Error bars represent standard deviations of measurements from triplicate cultures.

As we reported previously, the dark CO_2_ uptake peaks at the DHAL interfaces coincided with the recovery of metabolically active chemolithoautotrophic *Thaumarchaeota* belonging to MG1 and members of various proteobacterial classes (Yakimov et al., 2007, 2013, 2015; La Cono et al., 2011). As far as these organisms seem to act as the key players in highly active CO_2_ fixation processes detected at these depths, the samples of the *L'Atalante* and *Kryos* interfaces were used to obtain autotrophic and nitrifying enrichments ATA and KRY. The enrichment SAL5 was established using the bathypelagic sample (3,000 m depth), collected over the brine lake *Urania*. Ammonia consumption in the enrichment cultures was initially observed after 6 months of incubation at 16°C. Consecutive cell passages in NH_4_Cl-containing medium over a period of 2 years resulted in the stably nitrifying ATA, KRY and SAL5 enrichments. Independently on initially added amount, ammonia was completely disappeared in the cultures after 35–40 days of cultivations (data not shown), although no stoichiometric production of nitrite via ammonia oxidation was observed (Figure 3), likely indicating that ammonium was used both in dissimilation and assimilation processes. Specifically, the maximum amount of produced nitrite (384 ± 13 μM) was detected in the ATA enrichment after 35 days of incubation in the dark at 16°C. The SAL5 enrichment, amended with 100 μM NH_4_Cl, converted 84–87% of ammonia after 1 month of cultivation. The KRY enrichment was least active in nitrification and only 56% of available ammonia was converted in nitrite. As far as the scope of the present study was to obtain stable nitrifying and CO_2_-fixing enrichments, rather than the pure culture of ammonia-oxidizing chemolithotrophs, the presence of ammonia-assimilating bacterial population was not excluded. Hence, eventual heterotrophic members of consortia likely assimilated the residual ammonia. The subsample (5 mL) for the ABD measurements, was taken from the enrichments after 3 weeks of cultivation, which corresponded to a period when one half of the maximum nitrite was produced (Figure 3). Very fast bicarbonate assimilation rate (20.7 ± 1.5 ng C mL^−1^ d^−1^) has been detected in the ATA enrichment, which was twice as high the ABD rates, observed in the SAL5 enrichment (10.7 ± 0.9 ng C mL^−1^ d^−1^). Daily cellular specific rates of bicarbonate assimilation (csABD) varied depending on enrichments, although in much less extent (1.12–1.64 fgC cell^−1^ d^−1^). These csABD assimilation activity values, which coincide well with our abovementioned data, confirm the consistency of the present study.

### Phylogenetic analysis of archaeal component in the enrichment cultures and characterization of ammonia-oxidizing archaeal ecotypes

**Figure 4:**
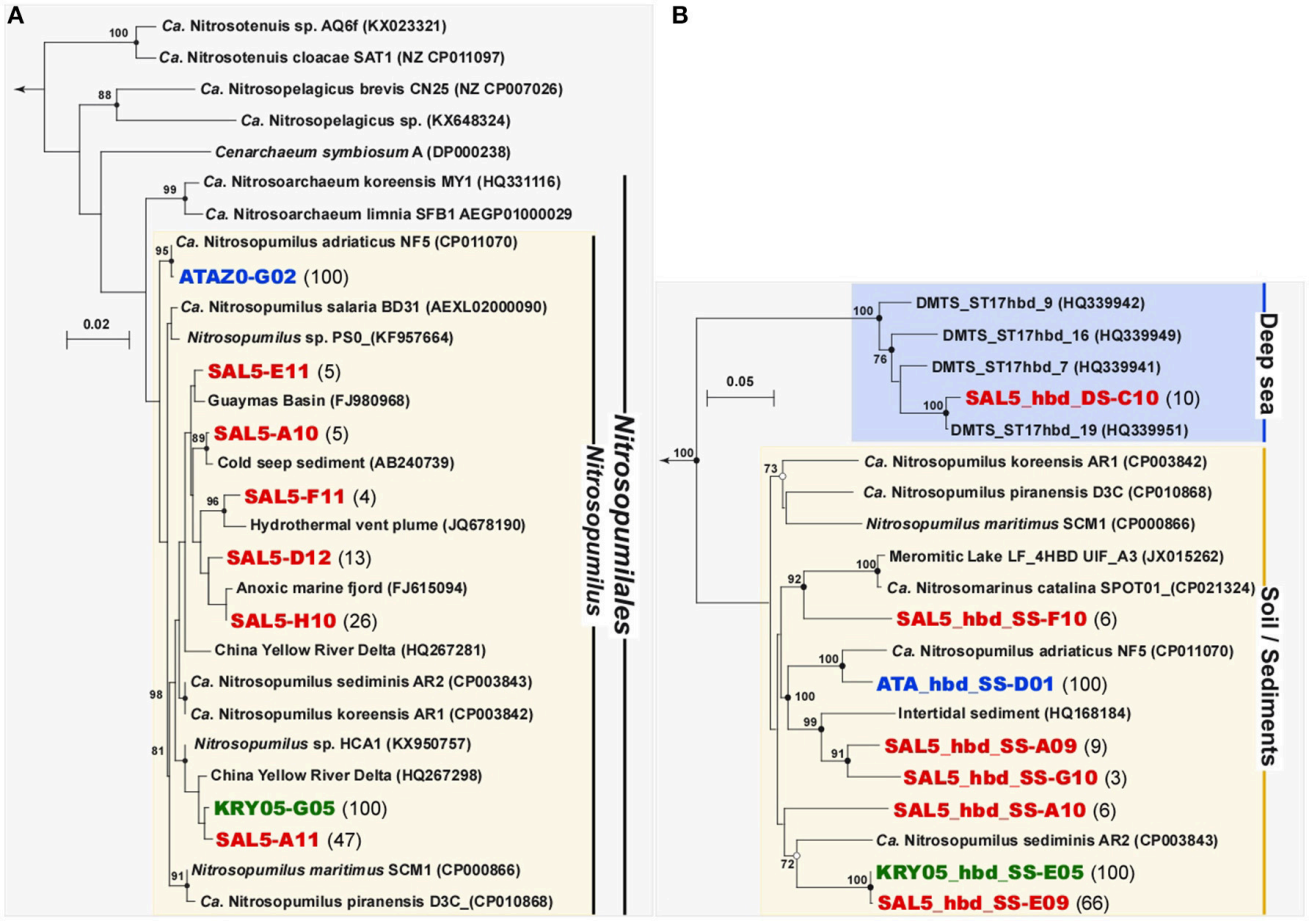
Phylogenetic affiliation of the archaeal 16S rRNA **(A)** and putative 4-hydroxybutyryl-CoA dehydratases 4-HBD **(B)** gene sequences, retrieved from three stably nitrifying enrichments ATA, KRY and SAL5. The tree was constructed using MacVector 11.1.2 software by Neighbor-Joining method and Jukes–Cantor distance matrix. Non-parametric bootstrapping was performed upon 1,000 iterations to infer tree topology and the nodes with the percentage of bootstrap re-sampling above 70% are indicated. The 16S phylogenetic tree was rooted with *Haloferax lucentense* 16S ribosomal RNA gene (AH003665), while the *4-hbd* tree was out-grouped with 3-hydroxybutyryl-CoA dehydrogenase of *N. maritimus* SCM1 (Nmar_1028, ABX12924). The frequencies of all phylotypes of 16S rRNA and *4-hbd* genes, present in clone libraries, are evidenced at the end of each sequence as percentage of analyzed clones. The scale bar represents the number of fixed mutations per nucleotide position.

Phylogenetic analysis of archaeal 16S rRNA clone libraries showed that all three enrichments were consisted exclusively of members of *Thaumarchaeota* Marine Group I.1a (provisional order *Nitrosopumilales*) belonging to the genus *Nitrosopumilus* (Figure 4). Noteworthy, both high ammonium concentration-amended ATA and KRY enrichments were inhabited by two different but monophyletic archaeal cultures. Based on 16S rRNA gene phylogeny, the ATA culture was identical (99.9%) to “*Ca*. N. adriaticus” NF5, enriched from Northern Adriatic coastal surface waters sampled off Piran (Slovenia) at the depth of 0.5 m (Bayer et al., 2016). Together with environmental clones, recovered worldwide from various coastal marine and estuary sediments, the KRY culture forms a sub-cluster, tightly related (99.2%) to the strain HCA1, enriched from 50 m water from the Puget Sound Regional Synthesis Model (PRISM) Station P10 in Hood Canal (Salish Sea, Pacific Ocean) (Qin et al., 2014). Surprisingly, the total of six *Nitrosopumilus* phylotypes (the similarity cutoff of <99%) were recovered from low ammonia-amended SAL5 enrichment. The most representative phylotype, SAL5_A11 (47% of all clones sequenced), was almost identical to the KRY culture KRY05-G05 (99.6% of identity), whereas remaining clones were grouped into five distinct groups, recovered from different deep-sea and oxygen-depleted marine ecosystems worldwide without cultivated representatives (Figure 4A).

All *Thaumarchaeota* that have been characterized thus far, possess a modified 3-hydroxypropionate/4-hydroxybutyrate (3HP/4HB) pathway, which has been proposed as the most energy-efficient aerobic pathway for CO_2_ fixation (Könneke et al., 2014). In addition to acetyl-CoA carboxylase, initiating the 3HP/4HB cycle, the second key enzyme, unambiguously indicating the presence of autotrophic 3-HP/4-HB pathway, is the 4-hydroxybutyryl-CoA dehydratase (4-HBD) (Berg et al., 2007, 2010). This FAD- and [4Fe−4S]-containing enzyme, which catalyzes dehydration of 4-hydroxybutyryl-CoA with production of crotonyl-CoA, is very conservative in mesophilic autotrophic *Thaumarchaeota*. Using the primer pair, specifically targeting thaumarchaeal *4-hbd* gene sequences (La Cono et al., 2010), we were able to amplify this gene from all enrichment cultures and a total of 32 archaeal *hbd* gene fragments were cloned and sequenced from each libraries. In strict accordance with observed 16S rRNA gene phylogenetic diversity, both high ammonium-amended enrichments were monophyletic, while SAL5 possessed six distinct phylotypes (Figure 4B). As in the case with 16S rRNA gene, the most representative cluster of SAL5 *4-hbd* clones (66% of all clones analyzed) were identical to monophyletic KRY phylotype. Noteworthy, the SAL5_hbd_DS-C10 phylotype (10% of all clones analyzed) were affiliated with the “Deep-sea Cluster” (Yakimov et al., 2011). Before our study, this cluster, consisting of exclusively bathypelagic sequences, did not possess any cultivated representatives.

Recently, Sintes et al. (2013, 2015, 2016) showed that phylogenetically distinct clusters of marine ammonium-oxidizing thaumarchaea (AOT) inhabit different water layers and regions, which are characterized by various ammonia availability. Consequently, they were divided into the high and low ammonia concentration AOT clusters (HAC- and LAC-AOT). HAC-AOT are dominating in the ocean regions with relatively high ammonia concentrations, like polar and epipelagic waters. In contrast, LAC-AOT prevail in deep-ocean environments, where ammonia concentrations is often below the detection limit of conventional methods. Following the most relevant classification, all sequences of the marine archaeal *amoA* genes are grouped within six main subclusters (Sintes et al., 2016). Two of them (subclusters 1 and 2) fall into the HAC-AOT cluster and include the sequences from Water Cluster A (WCA) or surface cluster (Francis et al., 2005; Hallam et al., 2006; Mincer et al., 2007). Most epipelagic archaeal *amoA* sequences affiliated to subcluster 2, whereas subcluster 1, which includes *N. maritimus* SCM1 *amoA*, is more heterogeneous and contains sequences from various marine compartments, including estuaries, lagoons and sediments (Sintes et al., 2016). Four remaining subclusters belong to the LAC-AOT and are dominated by sequences from deep-waters (>200 m depth).

**Figure 5:**
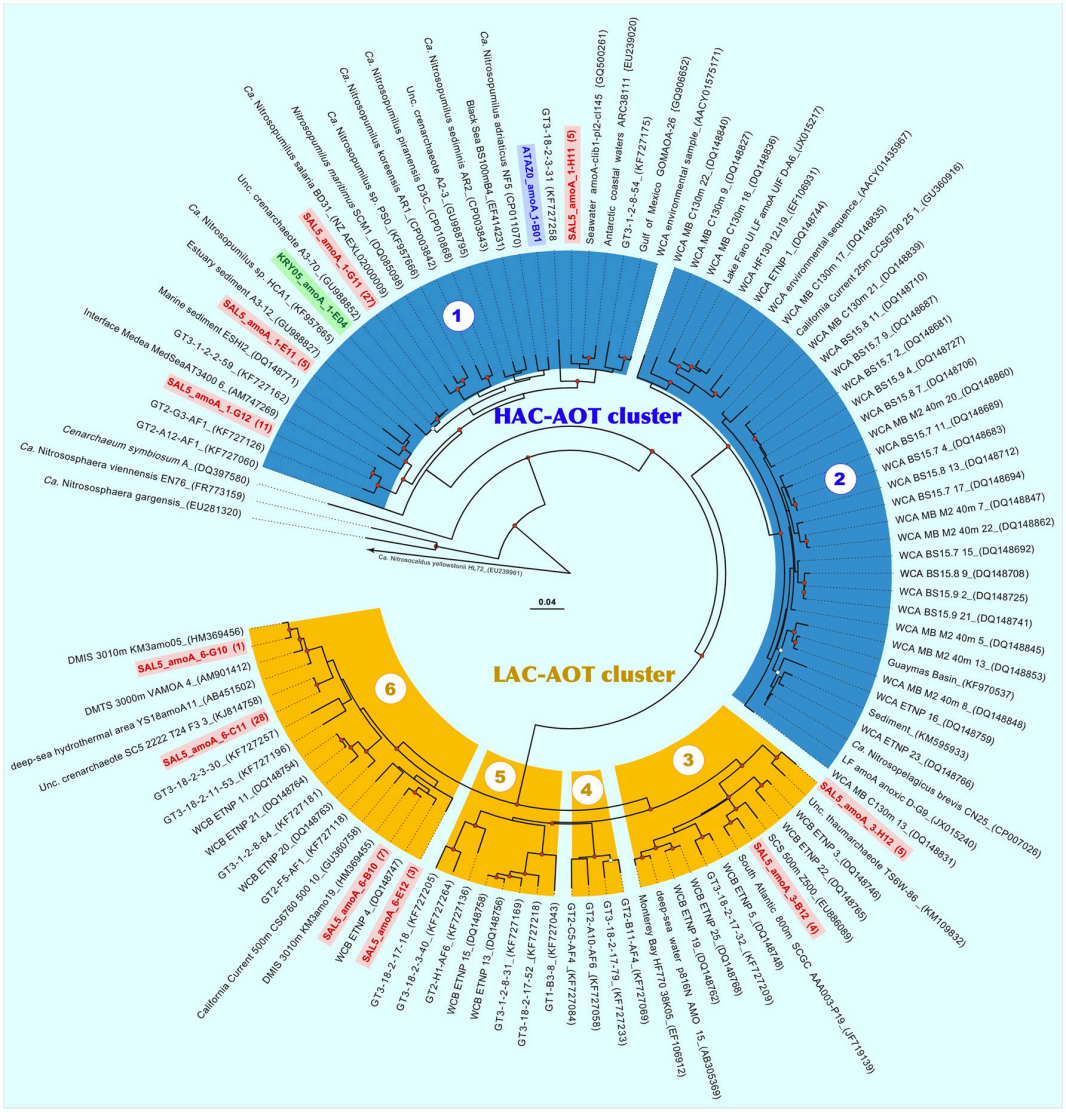
Neighbor-joining phylogenetic nucleotide tree of thaumarchaeotal *amoA* obtained from the three stably nitrifying enrichments ATA, KRY, and SAL5. The marine clades 1–2 of high ammonia concentration ammonium-oxidizing archaea (HAC-AOT) and the marine clades 3–6 of low ammonia concentration ammonium-oxidizing archaea (LAC-AOT), as defined by Sintes et al. (2016), are color-coded as aquamarine and yellow, respectively. Sequences of enrichments are also color-coded according to types of clone library origins, as blue (ATA), green (KRY), and red (SAL5). Significant bootstrap values are shown as open (>50) and red-filled (>70) circles at branch nodes.

Based on observed diversity of 16S rRNA and *hbd* genes in our enrichments, a total of 32 *amoA* gene fragments were cloned from the ATA and KRY enrichments, while 96 *amoA* clones were analyzed from the SAL5 library. As it was expected from phylogenetic analyses, all ATA and KRY *amoA* sequences were monophyletic and correspondingly affiliated to *Ca*. Nitrosopumilus adriaticus NF5 and *Nitrosopumilus* sp. HCA1 (Figure 5). Diversity of SAL5 *amoA* sequences was much more profound and at least 10 distinct phylotypes (identity cutoff of <97%) were recovered. Noteworthy, only half of SAL5 clones were belonged to subcluster 1 of the HAC cluster, whereas remaining clones were grouping into 6 phylotypes affiliated with members of subclusters 3 (9%) and 6 (41%) of the LAC *amo*A ecotype. Epipelagic HAC and bathypelagic LAC ecotypes of ammonium monooxygenase were recently distinguished at the amino acid level into 38 different oligotypes (Sintes et al., 2016). It has been suggested, that due to the low ammonium concentrations in deep waters, such amino acid substitutions in the AmoA monooxygenase might lead to various adaptive responses: (i) to increase in affinity toward the substrate; (ii) to broaden the spectrum of available substrates; or (iii) to the complete loss of the enzymatic activity due to a lower impact of ammonia oxidation on the *Thaumarchaeota* fitness in ammonium-impoverished environment. Using this classification, we confirmed that together with AmoA of *Nitrosopumilus* sp. HCA1 both KRY and two SAL5 phylotypes belonged to oligotype 12, while remaining phylotypes of HAC-*amo*A cluster (ATAZ0-1 and SAL5-1-G11) possessed novel amino acids combinations, unassigned to any of previously known oligotypes (Table S2). Among the LAC phylotypes, retrieved from SAL enrichment, the aa substitution SAL5-4-H12 was also coined as a novel oligotype. Noteworthy, only thaumarchaeotal representatives of the HAC-AmoA cluster have been enriched or isolated so far (Könneke et al., 2005; Santoro and Casciotti, 2011; Park et al., 2014; Qin et al., 2014; Santoro et al., 2015; Bayer et al., 2016), and it was indicated that more emphasis should be put on offering more realistic ammonium concentrations in culturing approaches than done hitherto (Sintes et al., 2016). Here, for the first time, we demonstrated that members of the LAC ecotype, adapted to low ammonia concentrations, can be obtained in laboratory and maintained as stable enrichments supplemented with 100 μM NH_4_Cl.

### Phylogenetic analysis of bacterial component in the enrichment cultures

**Figure 6:**
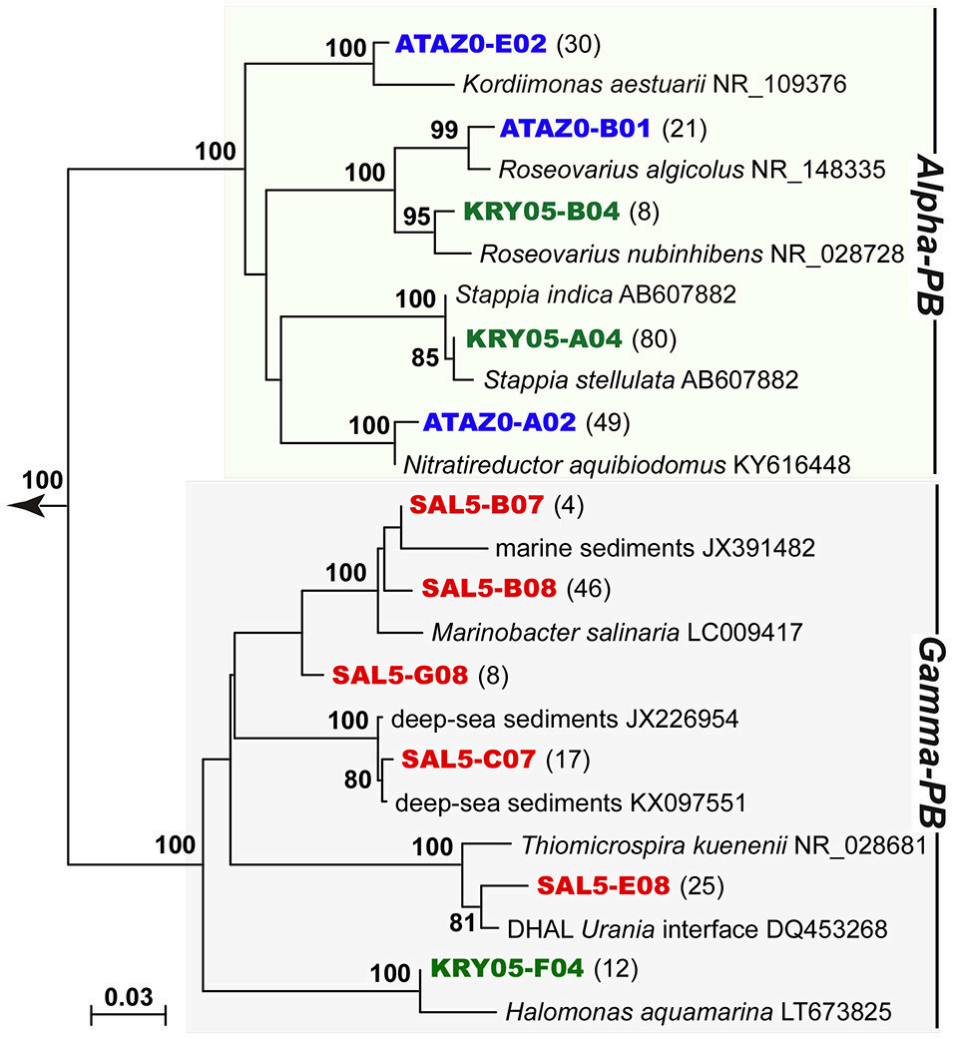
Phylogenetic affiliation of the bacterial 16S rRNA gene sequences, retrieved from the stably nitrifying enrichments ATA, KRY, and SAL5. The tree was constructed by Neighbor-Joining method and Jukes–Cantor distance matrix using MacVector 11.1.2. Non-parametric bootstrapping was performed upon 1,000 iterations to infer tree topology and the nodes with the percentage of bootstrap re-sampling above 70% are indicated. The 16S phylogenetic tree was rooted with *Sunxiuqinia elliptica* 16S ribosomal RNA gene (GQ200196). The frequencies of all phylotypes of 16S rRNA, present in clone libraries, are evidenced at the end of each sequence as number of corresponding clones, respectively. The scale bar represents the number of fixed mutations per nucleotide position.

Recent studies on DIC assimilation in aphotic ocean reveal a surprising diversity of marine bacteria actively performing this process (Varela et al., 2008; Alonso-Sáez et al., 2010; Lenk et al., 2011; Llirós et al., 2011; DeLorenzo et al., 2012; Yakimov et al., 2014; Dyksma et al., 2016; Cao et al., 2017). Different marine bacterial lineages are capable of DIC fixation, including putatively heterotrophic taxa of *Alpha*- and *Gammaproteobacteria*, which usually dominated the active DIC-assimilating prokaryoplankton communities worldwide. According to our nitrification assay, the phylogenetic analysis, performed on 96 clones from each library, revealed the co-occurrence in all three nitrifying enrichments of thaumarchaeal population and bacteria exclusively belonging to the *Alpha*- and *Gammaproteobacteria* (Figure 6). Within bacterial clones, the KRY population consisted mainly of the marine genus *Stappia* (75% of the total number of clones). In addition to the *coxL* gene for aerobic CO dehydrogenase, members of this genus often possess *cbbL*, the large subunit gene for ribulose-1,5-bisphosphate carboxylase/oxygenase (RuBisCO form I), suggesting their possibility of lithotrophic or mixotrophic metabolism (Weber and King, 2007). The genes coding for the large subunit of RuBisCO form I were found also in sulfur-oxidizing bacteria of genera *Thiomicrospira* and *Roseovarius*, amounting to 26 and 21% of the total SAL5 and ATA bacterial clones, respectively. The greatest number of SAL5 bacterial clones (56%) were classified within the genus *Marinobacter* known to persist under extremely oligotrophic and nutrient-limited conditions (Riedel et al., 2013) and to possess efficient aerobic denitrification ability (Zheng et al., 2012). Autotrophic denitrification performance was also reported for extremely oligotrophic bacteria belonging to the genus *Nitratireductor* (Nguyen et al., 2015; Park et al., 2017), which was represented by almost half of all ATA clones analyzed. Remaining clones of the ATA library belonged to genus *Kordiimonas*. Similarly to *Marinobacter* and *Nitratireductor*, enrichment and isolation of *Kordiimonas* strains usually require low nutrient media and long incubation periods (Wu et al., 2016). Observed co-occurrence patterns of *Thaumarchaeota* with chemoheterotrophic *Proteobacteria* are in coherence with cultivation and physiological assays recently performed on autotrophic AOT (Beman et al., 2011; Park et al., 2014; Qin et al., 2014; Bayer et al., 2016). It was suggested that in concert, these prokaryotic groups might be involved in the re-mineralization of organic material and hence, nutrient cycling. Additionally, the dependence of autotrophic AOT on small organic molecules as required metabolites provided by bacterial component of consortium cannot be completely excluded.

Taken together, our results provide evidence for complex interactions between various physiological groups of bathypelagic prokaryotes and point toward careful quantification of several key metabolic processes involved in the global carbon cycle/sink in the ocean interior. The long-term bicarbonate-assimilating and nitrifying enrichments, conducted in present study, indicate that both HAC and LAC ecotypes of ammonium-oxidizing *Thaumarchaeota* can co-occur stably with chemoheterotrophic *Proteobacteria*, hence supporting an assumption on their symbiotic interactions in natural environments. Notably, besides the amount of added ammonia, all enrichments were otherwise similarly treated. Nonetheless, the LAC-amended SAL5 enrichment exhibits consistent differences in AOT diversity, compared to the conventionally processed ATA and KRY enrichments. Although with the information available at the moment, we cannot determine the specific effect of added ammonia (e.g., loss of enzymatic function or eventual toxicity of intermediates), the strong positive selection toward the HAC ecotype in the enrichments, supplemented with 500 μM NH_4_Cl, might indicate some degree of environmental adaptation leading to niche specialization of these two main ecotypes of AOT. From these data, we can begin to pinpoint genomic adaptations of the ecologically important ubiquitous deep-sea LAC ecotype, and further understand their environmental constraints and metabolic potential.

## Author contributions

VL, LG, and MY conceived the research. VL, GR, FC, and RD did enrichment work. FS, GL, FD, LM, GM, MA, VL, FC, MY did oceanographic work and performed all the data analysis. MY drafted the manuscript, VL, FS, MA, and LG contributed to the manuscript writing.

### Conflict of interest statement

The authors declare that the research was conducted in the absence of any commercial or financial relationships that could be construed as a potential conflict of interest.
