# Supplementary material for: Contribution of Bicarbonate Assimilation to Carbon Pool Dynamics in the Deep Mediterranean Sea and Cultivation of Actively Nitrifying and CO2-Fixing Bathypelagic Prokaryotic Consortia
Source: Front Microbiol. 2018 Jan 19;9:3. doi: 10.3389/fmicb.2018.00003 (PMC5780414; doi:10.3389/fmicb.2018.00003)
Supplement: Supplementary file 1 [file Table1.doc]

**TABLE S1 | The rates of [^3^H]-leucine incorporation measured by micro-method over 9 hours of incubation as disintegrations per minute (dpm) in bathypelagic water (3,000 meters) of Station 7**.

| **Incubation, min** | **[^3^H]-leucine incorporation, dpm** | | | |
| --- | --- | --- | --- | --- |
|  | **Sample** | **SD, n=3** | **Blank 1** | **Blank 2** |
|  |  |  |  |  |
| 60 | 571 | 18 | 417 | 397 |
| 120 | 739 | 2 | 420 | 438 |
| 180 | 1081 | 179 | 564 | 580 |
| 300 | 1247 | 38 | 491 | 503 |
| 420 | 1467 | 98 | 491 | 461 |
| 540 | 1730 | 334 | 463 | 511 |

^a^ study performed on aphotic water column;

^b^ study performed on oxic / anoxic interface of the deep-sea hypersaline anoxic lakes (DHAL);

^c^ site, used for enrichment setting (see Materials and Methods)
