# Supplementary material for: Contribution of Bicarbonate Assimilation to Carbon Pool Dynamics in the Deep Mediterranean Sea and Cultivation of Actively Nitrifying and CO2-Fixing Bathypelagic Prokaryotic Consortia
Source: Front Microbiol. 2018 Jan 19;9:3. doi: 10.3389/fmicb.2018.00003 (PMC5780414; doi:10.3389/fmicb.2018.00003)
Supplement: Supplementary file 2 [file Table2.DOC]

**TABLE S2 | Amino acid residues for the different phylotypes / oligotypes recovered from actively nitrifying and CO_2_-fixing enrichment cultures ATA, KRY and SAL5.**

| **Phylotype** | **Position of aa residue in AmoA sequence** | | | | | | | | | | | | | | | | | | | | | **Oligotype^a^** |
| --- | --- | --- | --- | --- | --- | --- | --- | --- | --- | --- | --- | --- | --- | --- | --- | --- | --- | --- | --- | --- | --- | --- |
|  | **27** | **42** | **47** | **52** | **53** | **57** | **69** | **70** | **73** | **77** | **85** | **94** | **99** | **106** | **112** | **145** | **188** | **191** | **192** | **195** | **202** |  |
| **HAC-AmoA Cluster** | | | | | | | | | | | | | | | | | | | | | | |
| **KRY05-1** | **A** | **T** | **S** | **L** | **I** | **T** | **A** | **T** | **A** | **T** | **G** | **Y** | **T** | **A** | **V** | **T** | **A** | **A** | **C** | **A** | **N** | **12** |
| **SAL5-1-G12** |  |  |  |  |  |  |  |  |  |  |  |  |  |  |  |  |  |  |  |  |  |  |
| **SAL5-1-E11** |  |  |  |  |  |  |  |  |  |  |  |  |  |  |  |  |  |  |  |  |  |  |
| **SAL5-1-H11** | **A** | **T** | **S** | **L** | **I** | **C** | **A** | **T** | **A** | **T** | **G** | **Y** | **T** | **A** | **V** | **T** | **A** | **G** | **C** | **A** | **N** | **15** |
| **SAL5-1-G11** | **A** | **T** | **S** | **L** | **I** | **T** | **A** | **T** | **G** | **T** | **A** | **Y** | **T** | **A** | **V** | **T** | **A** | **G** | **C** | **A** | **N** | **UN^b^** |
| **ATAZ0-1** | **A** | **T** | **S** | **L** | **L** | **C** | **A** | **T** | **A** | **T** | **A** | **Y** | **T** | **A** | **V** | **T** | **A** | **G** | **C** | **A** | **N** | **Una** |
| **LAC-AmoA Cluster** | | | | | | | | | | | | | | | | | | | | | | |
| **SAL5-4-B12** | **S** | **V** | **A** | **T** | **I** | **V** | **A** | **T** | **G** | **Q** | **V** | **V** | **H** | **C** | **A** | **L** | **A** | **A** | **C** | **A** | **N** | **20** |
| **SAL5-6-B10** |  |  |  |  |  |  |  |  |  |  |  |  |  |  |  |  |  |  |  |  |  |  |
| **SAL5-6-E12** |  |  |  |  |  |  |  |  |  |  |  |  |  |  |  |  |  |  |  |  |  |  |
| **SAL5-6-C11** |  |  |  |  |  |  |  |  |  |  |  |  |  |  |  |  |  |  |  |  |  |  |
| **SAL5-6-G10** | **S** | **V** | **A** | **T** | **V** | **V** | **A** | **T** | **G** | **Q** | **V** | **V** | **H** | **C** | **A** | **L** | **A** | **A** | **C** | **A** | **N** | **22** |
| **SAL5-4-H12** | **S** | **V** | **A** | **T** | **V** | **V** | **G** | **A** | **G** | **Q** | **V** | **V** | **H** | **C** | **A** | **L** | **A** | **A** | **C** | **A** | **N** | **UN** |
|  |  |  |  |  |  |  |  |  |  |  |  |  |  |  |  |  |  |  |  |  |  |  |

| **AA characteristics** | Charged/Special |
| --- | --- |
|  | Polar |
|  | Hydrophobic |

^a^ Oligotypes, supporting the existence of different thaumarchaeal ammonia oxidizing ecotypes, were identified using recent classification of Sintes et al. (2016).

^b^ UN, unassigned.
